# Is Promiscuity Associated with Enhanced Selection on MHC-DQα in Mice (genus *Peromyscus*)?

**DOI:** 10.1371/journal.pone.0037562

**Published:** 2012-05-23

**Authors:** Matthew D. MacManes, Eileen A. Lacey

**Affiliations:** Department of Integrative Biology, Museum of Vertebrate Zoology, University of California, Berkeley, California, United States of America; North Carolina State University, United States of America

## Abstract

Reproductive behavior may play an important role in shaping selection on Major Histocompatibility Complex (MHC) genes. For example, the number of sexual partners that an individual has may affect exposure to sexually transmitted pathogens, with more partners leading to greater exposure and, hence, potentially greater selection for variation at MHC loci. To explore this hypothesis, we examined the strength of selection on exon 2 of the MHC-DQα locus in two species of *Peromyscus*. While the California mouse (*P. californicus*) is characterized by lifetime social and genetic monogamy, the deer mouse (*P. maniculatus*) is socially and genetically promiscuous; consistent with these differences in mating behavior, the diversity of bacteria present within the reproductive tracts of females is significantly greater for *P. maniculatus*. To test the prediction that more reproductive partners and exposure to a greater range of sexually transmitted pathogens are associated with enhanced diversifying selection on genes responsible for immune function, we compared patterns and levels of diversity at the Class II MHC-DQα locus in sympatric populations of *P. maniculatus* and *P. californicus*. Using likelihood based analyses, we show that selection is enhanced in the promiscuous *P. maniculatus*. This study is the first to compare the strength of selection in wild sympatric rodents with known differences in pathogen milieu.

## Introduction

Understanding how natural selection shapes adaptive variation in populations of free-living animals has long been a focus of evolutionary biology [1]–[3]. Genes of the Major Histocompatibility Complex (MHC) provide logical targets for such studies because of the relatively well-understood function of these loci, which code for proteins that participate in recognition of and response to foreign antigens [4], [5]. Although the extreme diversity characteristic of MHC loci is well documented, identifying the underlying bases for diversifying selection on these genes has been more challenging. While multiple studies have attributed such selection on MHC loci to behavioral phenomenon such as mate choice [6]–[9], others have linked extreme allelic diversity at these genes to enhanced pathogen exposure [10]–[12]. Distinguishing between these alternatives provides an important opportunity to explore interactions between adaptively significant genotypic variation and the dynamic and variable selective pressures acting on free-living vertebrates.

One aspect of a species’ biology that may significantly impact exposure to pathogens is its mating system, specifically the number of partners with which an individual typically mates during either a single round of reproduction or over the course of its lifetime. Relationships between sexual behavior and sexually transmitted disease have been extensively studied in humans from both empirical and theoretical perspectives [13]–[16]. These studies have revealed that the number of concurrent partners [17], [18] and the amount of time between sexual encounters [19] are critical in determining the overall population level of infection. For example, in populations characterized by extreme promiscuity (i.e. high concurrency), rates of sexually transmitted pathogens tend to be high [20], [21]. In contrast, members of populations characterized by lifetime monogamy (i.e., low concurrency) have a much lower risk of contracting sexually transmitted diseases. Despite logically compelling links between sexual behavior, rates of pathogen transmission, and selection on immunogenes, few analyses have undertaken a comprehensive exploration of these relationships. As a result, studies that explore directly interactions among behavior, pathogen exposure, and selection on MHC genes are required.

Mice of the genus *Peromyscus* provide an ideal opportunity to explore these relationships in natural populations of vertebrates. Because these animals have long been targets of study, many aspects of their behavior and ecology are known [22]–[28]. With regard to mating system, the genus includes promiscuous [29], [30] and polygynous [31] species, as well as two of the few mammalian species demonstrated to be socially and genetically monogamous [32], [33]. In central coastal California, *P. californicus*– which has been shown in multiple studies to exhibit lifetime social and genetic monogamy [34] – is sympatric across its range with the socially and genetically promiscuous *P. maniculatus* [29]. Consistent with this difference in mating system, the reproductive tracts of female *P. maniculatus* are characterized by significantly more diverse bacterial communities than are those of female *P. californicus* [35]. These pronounced differences in reproductive behavior and potential pathogen exposure among otherwise ecologically similar congeners offer a rare opportunity to explore the effects of differences in mating system – specifically, the contrast between monogamy and promiscuity – on selection on MHC genes.

To characterize the relationship between mating system and selection on MHC genes in Peromyscus, we compared patterns of allelic and genotypic diversity at the Class II DQα locus in *P. californicus* and *P. maniculatus*. Given that promiscuity in the latter species is associated with an increased diversity of vaginal bacteria [35], we predicted that the greater number of reproductive partners per individual in *P. maniculatus* would also be related to enhanced diversifying selection at the DQα locus. We tested this prediction using data obtained from sympatric populations of these species whose mating systems have been previously characterized. This study is one of the first to describe the relationship between mating system and selection on MHC genes in natural populations of vertebrates. The comparative approach employed is particularly compelling in that it minimizes the effects of environmental differences on selection at these loci. As a result, our analyses provide important new insights into the effects of selection apparent relationships between mating system and selection on MHC genes in free-living vertebrates.

## Materials and Methods

### Study Populations and Tissue Sampling

Field research was conducted on Landels-Hill Big Creek Reserve (36.011156°, -121.518644°), which is located approximately 30 km south of Big Sur, Monterey County, California, USA, during June-August 2009. Big Creek Reserve is a part of the University of California Natural Reserve System and consists of 15.57 km^2^ of coastal scrub, redwood forest, and oak woodlands. This locality was selected because both *P. maniculatus* and *P. californicus* are abundant and can be captured in the same habitats using a single trapping grid.

To facilitate handling, all animals captured were anesthetized with Isoflurane. After light anesthesia was induced, a 3 mm^3^ section of the distal portion of the right ear pinna was removed using sterile scissors. Tissue was stored in 70% ethanol and frozen at −20°C within 2 hours of collection. To avoid potential resampling of the same individuals, each animal was then individually marked using a small numbered ear tag (Monel 1005, National Band & Tag Company) placed in the remaining portion of the right pinna. All procedures were approved by the University of California, Berkeley Animal Care and Use Committee and were in accord with the guidelines of the American Society of Mammalogists [36].

### DNA Extraction and General PCR Procedures

We extracted genomic DNA from tissue samples from 20 individuals per species using a salt extraction method [37]; the resultant extract was tested for purity and concentration using the Nanodrop system (Thermo Scientific). From this DNA stock solution, 50 ng/uL dilutions were prepared for use in all PCR reactions.

### Comparisons with Neutral Markers

Neutral processes associated with differences in effective population size (e.g. genetic drift) can affect how genes respond to natural selection. To ensure that differences in effective population size were not responsible for observed differences in selection regime, we genotyped all individuals sampled at 8 previously identified microsatellite loci using PCR conditions described by Chirhart et al., [38] and Mullen et al., [39]. Genotyping was accomplished on an ABI 3730 using the size standard LIZ500; allele sizes were scored using the program GeneMapper. The resultant dataset was screened for null alleles, linkage and Hardy-Weinberg disequilibrium using the program Genepop [40], [41]. Summary statistics on variability were generated in the program Arlequin [42]. Effective population size was estimated with the program ONeSAMP [43]. The sensitivity of the results to the user-assigned upper and lower limits of N_e_ was assessed by analyzing the data using a variety of values; analyses were run using lower bounds of 2–20 and upper bounds of 30–100.

### MHC Genotyping

Polymerase chain reaction amplification targeting a 246 base pair region of exon 2 of the DQα locus was carried out using *Peromyscus*-specific primers [44]. Because direct sequencing of highly polymorphic nuclear genes can be confounded by heterozygosity as well as by both insertion and deletion mutations, successful MHC amplicons were TA cloned using a TOPO TA Cloning Kit for Sequencing (Invitrogen, K4575-01) following the manufacturers instructions. A minimum of 8 positive colonies per individual was PCR amplified using the vector specific primers M13R and M13F and conditions specified by the manufacturer. Successful PCR reactions were cleaned using ExoSap (USB Corporation, #78250) and cycle sequenced using ABI BigDye Terminator v3.1 Cycle Sequencing Kits (Applied Biosystems, #4337456). Clones were sequenced in both forward and reverse directions on an ABI3730 automated DNA sequencer. Sequences were edited and assembled using the program Geneious 5.53 [45]. This process included the removal of primer sequences as well as nucleotides falling below a specified quality threshold (PHRED <20).

The number of clones sequenced per individual was based on a simple probabilistic model assuming unbiased PCR amplification and recovery of alleles; under a binomial distribution, sequencing of 8 positive clones per individual should have detected both alleles in a heterozygous individual with a probability of 0.992. An individual was considered heterozygous only if each allele sequence was detected in at least 2 distinct clones. Additionally, sequences were screened for the presence of recombination using the GARD [49] and SBR [50] algorithms contained in the program HyPhy, as well as with the PERMUTE code contained within the program omegaMap [46].

To assess the potential amplification of pseudogenes, coding sequences were translated and checked for the presence of an open reading frame and absence of stop codons. In addition, we compared our sequences to those of closely related species contained in GenBank in an attempt to identify unexpectedly high levels of sequence divergence that could reflect amplification of non-coding pseudogenes. To assess the possibility of >2 alleles per individual (indicative of multiple copies of the DQα locus), we sequenced 24 clones containing the correct insert size from each of 8 randomly chosen individuals per species. Using the same reasoning outlined above, this procedure should have detected a 3^rd^ allele (indicative of multiple copies of the DQα locus) with a probability of 0.9999.

**Figure 1:**
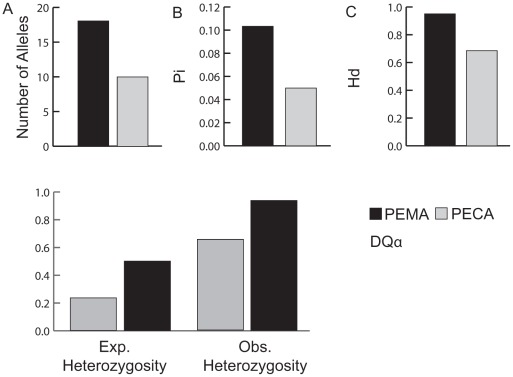
Comparisons of variability at the DQα locus. A. Number of alleles detected for each species. B. Estimated values for Pi for each species. C. Estimated values for haplotype diversity for each species. D. Observed and expected values of heterozygosity in both species. For both species, Hardy-Weinberg tests resulted in values of P = 0.000. In all cases, indices are greater for *P. maniculatus* than for *P. californicus*.

### Tests of Neutrality and Gene Diversity

Estimates of Tajima’s D, Fu’s Fs, Pi and haplotype diversity were calculated using the program Arlequin [42] and plotted in Fig. 1. The first 2 of these statistics use the site-frequency spectrum of nucleotide polymorphism to test for departures from neutrality [49], [50]. Tajima’s D and Fu’s Fs were calculated for the whole exon in an attempt to identify deviations from neutral expectations. In addition, the McDonald-Kreitman [47] test of neutrality was attempted in the program DNAsp [48], but no test statistic was calculated as the dataset lacked fixed differences between study species. Observed and expected heterozygosities were calculated and heterozygote deficiency was assessed using the Hardy-Weinberg Exact test implemented in Genepop.

### Alignment and MHC Gene Tree Construction

Because many algorithms for the detection of selection are based on comparative phylogenetic methods [51], [52], gene trees were constructed for DQα. The outgroup species consisted of a single DQα allele from 2 rodents–*Cricetelus barabensis* (FJ209306) and *Microtus arvalis* (DQ137813). Alignments were constructed using the program Muscle [53]. Sequences were partitioned by codon position and analyses were run in MrBayes [54], using the HKY85 model of molecular evolution, until model convergence was reached. A 50% consensus tree was built after discarding the first 1/3^rd^ of parameter values and trees as burn in.

### Analyses of Selection

To examine evidence of selection on the DQα locus, we implemented two distinct approaches. First, the subroutine codeml of the program PAML4.5 [51] was run. PAML employs a classic, phylogenetically-based maximum likelihood approach to detect the signature of natural selection using a series of likelihood ratio tests. Specifically, this procedure compares a series of models that allow omega ( = ω, equivalent to d_N_/d_S_) to vary between sites but not between lineages. Four independent models of molecular evolution were used to construct two likelihood ratio tests. M1a (Nearly Neutral) allows 2 site classes, one with ω = 1 and one with 0<ω<1. This model is compared with M2a, which is identical to M1a, but with the addition of another site class where ω>1. M7 (beta) assumes a beta distribution (limited to 0<ω<1) of codon sites, and is compared to M8, which is identical to M7, but with the addition of another site class where ω>1. For each test (M1a vs. M2a and M7 vs. M8), log likelihood values were calculated and compared using a likelihood ratio test.

**Figure 2:**
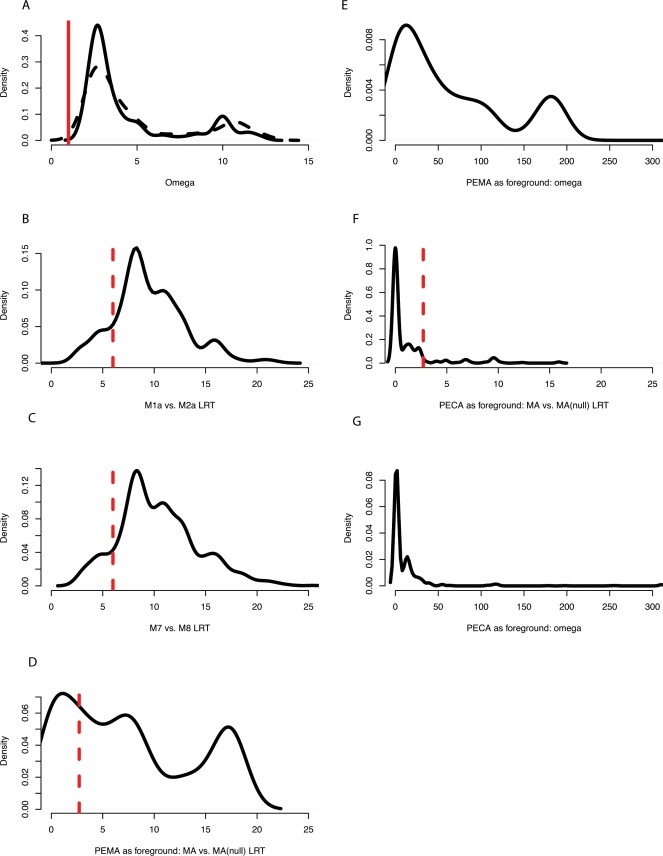
Plots of the distribution of selection parameters and statistical tests generated in the program PAML using the 1000 replicate datasets. Figure 2A is the distribution of omega. Dashed line is the estimate in model 2a, while the solid lines represent the estimate in model 8. 2B, 2C, 2D, 2F are the distributions of the LRT for M1a vs. M2a, M7 vs. M8, branch site tests (PEMA foreground) and branch site test (PECA foreground) respectively. The dashed red line indicates the critical value with df = 2, while the solid red line is placed at omega = 1. Figure 2E and 2G plot the distribution of omega in the branch site test when PEMA or PECA are placed in the foreground position.

Because we were interested in using PAML (typically employed for inter-specific comparisons) as described above on our population level dataset, we randomly selected a single allele from each focal species for analysis of natural selection using a python script that employed a random sampling with replacement strategy. We repeated this jack-knifing sampling procedure 1000 times, creating 1000 datasets each containing 4 sequences (two focal species, two outgroup species), thus ensuring that all results were robust to the effects of specific alleles or combinations of alleles. A tree was constructed as described above for each of the 1000 four-species alignments, after which this tree was used for the PAML analyses (1000 datasets x 4 PAML models). The distribution of omega, the results of the likelihood ratio tests constructed for each of the 1000 replicates, and the results of the two tests (M1a vs. M2a and M7 vs. M8) were plotted (Fig 2a, b, c).

A major shortcoming of PAML is its inability to account for the effects of recombination when identifying signatures of natural selection. Because high levels of recombination can be associated with a high false-positive rate [55], we chose to analyze our data in an additional framework, where recombination, if present, could be explicitly considered. The program omegaMap uses a population genetic approximation to the coalescent and treats uncertainty in gene genealogy as a nuisance parameter, all within a Bayesian framework that results in inferences that are robust to recombination, making this approach particularly useful for the analysis of datasets like our population level samples of DQα variability.

Within omegaMap, we calculated selection parameters and identified specific codons thought to be subject to enhanced selection. All analyses were comprised of two separate MCMC chains of 1 million generations in length, with sampling from the likelihood surface every 100^th^ generation. Settings recommended in the software documentation were used, except for omega, which was modeled as either a constant variable when calculating a global estimate of omega, or as an independent variable when estimates of selection in specific codons were sought. Separate analyses were run for *P. maniculatus* and *P. californicus*. These two independent runs were compared to each other to assure that model convergence had been reached.

### Comparing Selection Across Study Species

To determine if selection has acted differently in *P. maniculatus* relative to *P. californicus,* the branch site model (model A) of diversifying selection [56], [57] was implemented within PAML. This model allows the user to partition the action of selection between a foreground lineage, where selection is hypothesized to be enhanced relative to a set of background lineages. The model is tested in the LRT framework against the null model in which omega is constrained to acting with equal strength in foreground and background lineages. For this part of the study, as part of separate program executions, we set either the monogamous *P. californicus* or the promiscuous *P. maniculatus* as foreground lineages. Because the dataset consisted of multiple alleles per species, we used an approach identical to that described above, randomly selecting an allele from each species (as well as the two outgroup species) and repeating this procedure to create 1000 datasets per species which were then run in the program PAML. Results of the LRTs for each test as well as the distribution of foreground omega values were plotted (Fig. 2d, e, f, g).

## Results

### Diversity of Microsatellite Markers

Variation at the 8 microsatellite markers examined was consistently greater for *P. maniculatus*. Mean number of alleles per locus for *P. californicus* was 7.7 (range 6–11) while the mean for *P. maniculatus* was 10.2 (range 7–14). Mean heterozygosity was 0.80 and 0.88, respectively.

Estimates of effective population size based on the microsatellite data revealed that N_e_ did not differ markedly between the study populations. The median effective population size for *P. maniculatus* was 24.2 (95% CI 21.5–27.5); for *P. californicus*, this value was 21.5 (95% CI 19.1–24.1). A comparable magnitude of difference was obtained regardless of the specific upper and lower bounds of N_e_ used for estimation.

### Diversity at MHC-DQα

As with the neutral markers examined, interspecific differences in diversity were evident for MHC-DQα. In total, 31 distinct DQα alleles were detected (Genbank JN703316-JN703377). Specifically, 18 alleles were detected for *P. maniculatus* and 14 alleles were detected for *P. californicus*; one of these alleles was shared between the study species. In no case did we find evidence of more than 2 alleles per individual for the DQα locus. Effectively no evidence of recombination was found in our dataset. In the program HyPhy, 96 and 97 potential breakpoints (points of recombination) were analyzed (GARD and SBR tests, respectively). No significant improvement in cAIC scores was observed when comparing a model that included a recombination event at any breakpoint over a model lacking recombination. Additionally, the PERMUTE code within omegaMap revealed no evidence of recombination in *P. maniculatus*, although a single test – the negative correlation between LD (measured as r^2^ [58]) and physical distance – did suggest a recombination event (p = 2.5×10^−5^) in *P. californicus*.

The number of alleles, Pi, haplotype diversity, and expected and observed heterozygosity were greater for *P. maniculatus* (Fig. 1). Thus, similar to the neutral microsatellite markers examined, diversity at DQα was generally greater for the promiscuous *P. maniculatus*. For both study species, Hardy-Weinberg tests revealed significant heterozygote excess at exon 2 of the DQα locus (both p<0.001). However, neither Tajima’s D nor Fu’s Fs provided evidence of significant departures from neutral expectations in either species (PECA: D = −0.79, Fs = 2.8; PEMA: D = 0.31, Fs = 1.6, all p>0.05).

Analyses of nucleotide variation at the DQα locus revealed evidence of selection on this gene. Specifically, likelihood ratio tests of models M1a versus M2a and M7 versus M8 suggested that diversifying selection has strongly influenced patterns of molecular diversity at the DQα locus. Regarding the comparison between M1a and M2a, the null model of neutral evolution was rejected in 870 of the 1000 tests with p<0.05 (Fig. 2b). For the comparison M7 vs. M8, the null model was rejected in 899 of 1000 tests with p<0.05 (Fig. 2c). The distribution of omega calculated using the 1000 replicate datasets was plotted (Fig. 2a). The mean omega calculated using M2 was 4.92, while the mean using M8 was 4.49. Using omegaMap, the posterior probability of positive selection in PEMA was.87 (omega = 1.29), while the in PECA it was.86 (omega = 1.40). The proportion of sites under significant diversifying selection (ω>1, Bayesian posterior probability >.95) was 0.207 in *P. maniculatus* and 0.146 in *P. californicus* (Table 1). In contrast, when the consensus sequence created for each species was used, no test in either software package demonstrated significant evidence of selection. We believe that this result is likely related to the analytical process through which the consensus is created (collapsing variation at individual nucleotides). Although for the majority of sites there is little effect, this process may blunt the signal at very variable, positively selected sites.

**Table 1:** Estimates of diversifying selection on exon 2 of the MHC DQα locus.

	Gene	n	Nc	dN/dS	Selection parameters	Pos. Seln omegaMap
**PEMA**	DQα	18	82	1.29 (.82–1.99)	p_s_ = .207	HPD = .87
					ws = 14.6	
**PECA**	DQα	14	82	1.40 (.76–2.53)	p_s_ = .146	HPD = .86
					ws = 29.1	

PEMA = *P. maniculatus*, PECA = *P. californicus*. n = number of alleles recovered, N_c_ = number of codons, selection parameters. d_N_/d_S_ averaged across all sites. w_s_ = omega at the proportion of sites under diversifying selection, p_s_ = the proportion of sites under selection. Pos. Seln. Indicates the Bayesian posterior probability that positive selection is occurring.

Branch site tests of lineage-specific selection strongly supported the hypothesis of enhanced selection in promiscuous species. When assigning the promiscuous *P. maniculatus* to the foreground lineage, 745 of the 1000 datasets run showed strong evidence of lineage-specific selection (Fig 2d), with selection enhanced in the promiscuous species at p<0.05. In contrast, when setting the monogamous *P. californicus* as the foreground lineage, less that 5% of all tests were significant (Fig. 2f). The distribution of omega in foreground lineages differed markedly between analyses, with a large proportion of the tests resulting in omega >1 with promiscuity in the foreground (Fig. 2e) versus a very small proportion of tests with this value when monogamy in the foreground (Fig. 2g).

Codon level evidence of selection–Analyses of codon level variation revealed 19 codons (n = 17 for *P. maniculatus*, n = 12 for *P. californicus*) that were identified by omegaMap as being subject to significant diversifying selection (Bayesian posterior probability >.95). Eight of these codons were identified as subject to significant selection in both study species (Table 2), all of which are known peptide binding sites in other mammalian species [59].

**Table 2:** Codons in exon 2 of the DQα locus inferred to be under significant positive selection in each study species.

Codon Site	Species		Function
	PEMA	PECA	
**8**		10.1	
**9**	16.0	24.7	PBR
**10**	6.1		
**22**	9.3	35.4	PBR
**29**	14.0	16.3	PBR
**40**	5.3		
**50**	45.3	49.9	PBR
**56**	15.9	66.8	
**59**	8.7		
**60**	5.2	11.9	PBR
**63**	10.2		PBR
**64**	30.0	47.7	PBR
**66**	12.9		PBR
**67**	10.9		PBR
**69**		5.1	
**70**	6.0		PBR
**71**	36.5	44.2	PBR
**72**	9.4	13.1	
**77**	6.8	24.3	PBR
			

Numbers represent values for d_N_/d_S_ inferred by omegaMap. Codons experiencing significant positive selection in both species are highlighted by red numbers. PBR indicates sites that are thought to directly interact with pathogens.

## Discussion

A growing body of literature suggests that the evolution of MHC genes is influenced by a complex suite of selective [60]–[62] and neutral processes [63]–[65]. This complexity makes it challenging to infer the underlying reasons for patterns of MHC variation, particularly in natural populations. A priori, we had predicted (based upon interspecific differences in mating system and diversity of vaginal bacteria) that diversifying selection should be stronger in the promiscuous *P. maniculatus*. Indeed, our analyses strongly support this hypothesis. For the branch site tests, both the number of significant tests and the distribution of omega in the foreground lineage were much larger when the promiscuous *P. maniculatus* was identified as the foreground species, suggested enhanced selection on DQα in this species compared to the monogamous *P. californicus*. This result is also supported by codon-level analyses, in which more codons were subject to diversifying selection in *P. maniculatus* than in *P. californicus*. Taken together, these outcomes provide convincing evidence that diversifying selection on the DQα locus is enhanced in the promiscuous *P. maniculatus*.

Although several methods have been proposed for determining whether recombination has altered the signal of natural selection in DNA sequence data [46], [55], [66], analyses of population-level data that may be subject to recombination remain challenging. In our dataset, only weak evidence for recombination was detected. Whether this represents a lack of statistical power or the absence of biologically relevant recombination is unclear. Despite this outcome, we believe that our analyses are robust to the effects of recombination. The outcomes of all tests for selection, including omegaMap (which explicitly considers recombination) and the LRT (M7 vs. M8) conducted within PAML (reported to be more robust to recombination than other tests in PAML [55]) were concordant in identifying enhanced selection in the promiscuous study species. Given that recombination, which has been purported to be generator of allelic diversity [67], [68], was only weakly evident in the background lineage for our branch site tests, we believe that the results of our analyses were not confounded by recombination and are instead indicative of the relative intensity of selection on the two study species.

Selection on specific codons–Codon-level analyses revealed that 30% more codons were under significant selection in *P. maniculatus* than in *P. californicus*, suggesting that selection on the DQα locus is enhanced in the promiscuous species relative to its monogamous congener. Fifteen codons in exon 2 of the DQα locus are thought to interact directly with antigens [59]; our analyses indicated that 12 of these codons were subject to diversifying selection in at least one of our study species. This concordance between sites that were subject to selection and the presumed functional role of these codons suggests that selection on immune response has contributed to variation at the DQα locus in our study animals. Interestingly, the specific codons identified as being subject to selection differed between the study species. Possible explanations for this variation include interspecific differences in codon function [69] and response to a species-specific pathogen community [70]. Use of emerging genomic tools such as site-directed mutagenesis should help to elucidate the reasons for these differences in the specific DQα codons subject to diversifying selection in *P. maniculatus* and *P. californicus*.

The role of demography–Natural selection occurs against the backdrop of demography and thus demographic parameters may play a critical role in shaping selection on MHC genes. Both historical (e.g., population expansion) and contemporary (e.g., dispersal) demographic processes can alter the nature of selection [71], [72]. Additionally, differences in effective population size (N_e_) can influence the strength of natural selection [63]–[65]. As a result, demography must be considered when comparing patterns or estimates of selection for different populations. In our study, effective population size was similar for the two study species. Nevertheless, other demographic factors may skew the signal of natural selection in these animals, including differences in mating system [73], [74]. In particular, restricting reproduction to pair bonded individuals - a characteristic of monogamous species like *P. californicus* - is expected to lower effective population sizes relative to non-monogamous species [75]–[77], which may result in a ‘blunting’ of the effects of selection, or even a scenario in which selection operates within the context of relaxed constraint. Interestingly, we did not detect significant departures from neutrality using standard population genetic tests (e.g. Tajima’s D and Fu’s Fs); evidence of selection was detected only when using divergence-based analyses (omegaMap and PAML). Although the reason for this discrepancy in outcomes is unknown, we suspect that this disparity is related to the way in which each test incorporates signals of the historical demography of a population.

Potential environmental effects–Environmental factors are thought to play a significant role in shaping MHC variation, including spatial variation in MHC polymorphism [78]–[81]. By focusing on sympatric populations of mice, our study sought to minimize the effects of environmental differences on pathogen exposure. The study species did, however, vary markedly with respect to mating system. The greater number of reproductive partners per individual in *P. maniculatus* was associated with a greater diversity of vaginal bacteria in females of this species [35]. Number of reproductive partners is not the only aspect of reproductive behavior that may influence pathogen exposure and thus selection on MHC loci. For example, although contacts with reproductive partners are expected to be more numerous in *P. maniculatus*, these contacts are believed to be brief, with no extended periods of close proximity or nest sharing by adults. In contrast, *P. californicus* form extended male-female pair bonds, with members of a pair nesting together throughout the year [82], [83]. Time spent with conspecifics influences an animal’s risk of infection by some pathogens [84], [85] and thus even if exposure to sexually transmitted pathogens is reduced in the monogamous *P. californicus*, exposure to pathogens with other modes of transmission may be enhanced due to the prolonged contact between paired individuals. Although the analyses presented here do not address the relative importance of sexual versus social transmission, the finding that selection is enhanced in promiscuous species (e.g. [86]) may shed light on the relative importance of these distinct modes of pathogen transmission. Future work that quantifies socially transmitted pathogens in our study species will clarify the impacts of sexual versus social contact on pathogen exposure and selection on MHC loci.

To assess the generality of our findings, comparative tests of the relationship between promiscuity and selection on immunogenes in mammals should be performed. Opportunities for such comparative studies, however, are limited since the necessary contrast between promiscuity and monogamy in closely related species is rare, given that monogamy is thought to occur in less than 5% of mammalian species [87]. Further, a truly replicate study would control for environmental exposure (i.e. sympatry), a factor that we believe is important when working with natural populations. Although laboratory studies of the relationship between mating system and selection on immunogenes are possible, both important behavioral parameters (e.g. mating behavior) and pathogen exposure are likely to vary between wild and captive animals [88], thereby likely altering the relationship under analysis. Future work may begin with the systematic identification of monogamous species whose congeners are both closely related and promiscuous. Although the distribution of mammalian monogamy suggests that these comparisons are rare, researchers interested in determining the generality of our finding may find them important foci for the study of the relationship between sexual behavior and selection on immunogenes.
